# Allergy-associated biomarkers in early life identified by Omics techniques

**DOI:** 10.3389/falgy.2024.1359142

**Published:** 2024-02-23

**Authors:** Elisa Zubeldia-Varela, María Dolores Ibáñez-Sandín, Cristina Gomez-Casado, Marina Pérez-Gordo

**Affiliations:** ^1^ Institute of Applied Molecular Medicine Nemesio Díez (IMMA), Department of Basic Medical Sciences, Facultad de Medicina. Universidad San Pablo-CEU, CEU Universities, Madrid, Spain; ^2^ Department of Allergy, H. Infantil Universitario Niño Jesús, FibHNJ, ARADyAL- RETICs Instituto de Salud Carlos III, IIS-P, Madrid, Spain; ^3^ Department of Dermatology, University Hospital Duesseldorf, Heinrich-Heine University, Duesseldorf, Germany

**Keywords:** atopy, atopic dermatitis, biomarkers, early life, food allergy, metabolome, microbiome, proteome

## Abstract

The prevalence and severity of allergic diseases have increased over the last 30 years. Understanding the mechanisms responsible for these diseases is a major challenge in current allergology, as it is crucial for the transition towards precision medicine, which encompasses predictive, preventive, and personalized strategies. The urge to identify predictive biomarkers of allergy at early stages of life is crucial, especially in the context of major allergic diseases such as food allergy and atopic dermatitis. Identifying these biomarkers could enhance our understanding of the immature immune responses, improve allergy handling at early ages and pave the way for preventive and therapeutic approaches. This minireview aims to explore the relevance of three biomarker categories (proteome, microbiome, and metabolome) in early life. First, levels of some proteins emerge as potential indicators of mucosal health and metabolic status in certain allergic diseases. Second, bacterial taxonomy provides insight into the composition of the microbiota through high-throughput sequencing methods. Finally, metabolites, representing the end products of bacterial and host metabolic activity, serve as early indicators of changes in microbiota and host metabolism. This information could help to develop an extensive identification of biomarkers in AD and FA and their potential in translational personalized medicine in early life.

## 1 Introduction

The prevalence and severity of allergic diseases have increased over the last 30 years (1). The pathophysiological mechanisms underlying allergic diseases are different depending on the organs affected, but many patients who initially had an allergic disorder, such as atopic dermatitis (AD), eventually develop other allergies such as rhinitis, allergic asthma and/or food allergy (FA). This is commonly known as the “Allergic/ Atopic March” and refers to the natural history of allergic inflammation (2–4).

Atopic dermatitis, also known as eczema, is a skin disorder characterised by skin inflammation, with lesions and intense itch. It affects up to 30% of children and persists in 10% of adults in Western countries (5, 6). Skin barrier disruption could be the first step in the atopic march as well as in AD, leading to both skin inflammation and allergic sensitization. Although it is difficult to diagnose because of its great variety of symptoms, course, and severity, skin lesions, intense pruritus, and a chronic or relapsing course are used as AD diagnosis criteria. However, infants often present with poorly defined erythema lesions with oedema, excoriations, vesicles, and serous exudate, typically distributed on the face, cheeks, and trunk (7). Therefore, AD diagnosis and treatment mostly rely on clinical scores (8).

Food allergy is an unfavourable immune reaction towards food antigens, becoming a global health concern, since it affects both industrialized and developing countries, where their populations are adopting a westernized lifestyle (9). The estimated prevalence of FA is 8% in children and 5% in adults (10). It can be IgE-mediated and non-IgE-mediated. Non-IgE-mediated FA has a late onset of symptoms, and mostly affects the gastrointestinal system and skin, whereas IgE-mediated FA is characterized by a quick onset of symptoms, ranging from mild to severe life-threatening conditions, and involve the respiratory, gastrointestinal, dermatological, and cardiovascular systems (11). The complexity of FA makes it essential to diagnose, monitor and manage this disease by a personalized approach. In children, the diagnosis of FA has fairly improved in the last years, being clinical history, skin prick test and serum allergen-specific IgE levels, together with oral food challenge, the main diagnostic methods. In contrast, the management of FA needs improvement as food avoidance remains the major treatment nowadays.

A major challenge in current allergology is to unravel the mechanisms responsible for these diseases, in order to provide the patients with preventive, and personalised strategies, which has been collectively named as precision medicine (12). Biomarkers are genes, proteins, metabolites, or features by which a particular pathological or physiological process can be identified. They are valuable tools for precision medicine, as they can shed information for diagnosis and patient stratification, identification of therapeutic targets, and monitoring treatment efficacy (13–16). Identifying biomarkers of allergic diseases in early life could improve allergy handling at early ages and pave the way for personalized preventive and therapeutic approaches. Over the past few decades, high-throughput omic techniques have been developed and used in the search for allergy biomarkers (13, 17–19).

In this review, we summarize the current knowledge on three biomarker categories (proteome, microbiome, and metabolome) in two major allergic diseases such as AD and IgE-mediated FA in early life (20–23) (Table 1).

**Table 1:** Biomarkers associated to AD and/or FA in early life identified by omics techniques. Each omic technique is highlighted in different color: green for proteomics; red for metabolomics; blue for microbiome sequencing (genomics).

Pathology	Matrix	Compound/ Microorganism	Omic technique	Description	References
AD	Vernix	Polyubiquitin-C19; Calmodulin-like protein 5	Proteomics (LC-MS/MS)	Polyubiquitin-C and calmodulin-like protein 5 negatively correlated with AD	(24)
	Plasma from umbilical cord blood and infant blood	Interleukins, growth factors, chemokines and metalloproteinases	Proteomics (multiplexed affinity-based assay)	MHC class I proteins, IL-15, TNFAIP3 and MYDGF negatively correlated with AD; IL-9, IL-17, CCR10, GADD45A, MPO and MMP9 positively associated with AD later in life	(25)
	Serum	Interleukins, chemokines and extracellular metalloproteinases	Proteomics (OLINK)	inflammatory markers positively associated with AD lesional and non-lesional skin	(26)
	Skin swabs	Commensal *Staphylococci*	Bacterial 16S ribosomal RNA sequencing & Metagenomic sequencing	Commensal *Staphylococci* at 2 months of age are negatively associated with AD later. *S. epidermidis* significantly increased during AD flares. *S. epidermidis* predominates in patients with less severe AD	(27)
					(28)
					(29)
	Skin swabs	*Staphylococcus aureus*	MALDI-ToF MS & Metagenomic sequencing & Bacterial 16S ribosomal RNA sequencing	*S. aureus* at 3 months of age is positively associated with AD later. *S. aureus* prevalence at AD onset and 2 months before is higher than in healthy infants. At AD onset, infants with *S. aureus* were younger than uncolonized subjects. *S. aureus* predominates in patients with more severe AD. *S. aureus* correlated with more severe AD and during disease flares	(30)
					(29)
					(28)
	Skin swabs	*Streptococcus, Propionibacterium, Corynebacterium*	Bacterial 16S ribosomal RNA sequencing	*Streptococcus, Propionibacterium, Corynebacterium* increased during AD therapy	(28)
	Faeces	Fecal microbiota	Bacterial 16S ribosomal RNA sequencing & Quantitative PCR & Pyrosequencing	Reduced diversity is associated with AD. *Clostridium sensu stricto* is associated with AD// Clostridia at 5 and 13 weeks of age is associated with AD// *Clostridium* and *Akkermansia* negatively correlated with persistent AD. *Streptococcus* positively correlated with AD score (SCORAD)	(31)
					(32)
					(33)
					(34)
	Skin tape strips	Ceramides, Sphingomyelin	Metabolomics (lipidomics by LC-ESI-MS/MS)	Ceramides negatively correlated with *S. aureus* colonization. Sphingomyelin positively associated with AD	(35)
					(36)
					(37)
	Placenta	Glutathione thiol-disulfide balance	Metabolomics	Prenatal maternal depression and anxiety increase the risk of AD in offspring by decreasing the ratio of placental glutathione to glutathione disulfide	(38)
	Serum	Glutathione thiol-disulfide balance	Metabolomics (automated spectrophotometric method)	The thiol-disulfide balance in the patient group was weakened, and it shifted to the oxidative side in infants with AD.	(39)
	Plasma	PUFAs	Metabolomics (LC-MS)	Omega-3 polyunsaturated fatty acids (PUFAs) negatively and omega-6 PUFAs positively associated with AD later in life	(40)
	Urine	Lactate	Metabolomics (NMR spectroscopy)	Lactate was found to be increased in urine samples from children with AD	(41)
	Nutritional supplementation	Micronutrients	Logistic regression	Maternal intake of iron, folic acid, beta-carotene, vitamin E, vitamin D, zinc, calcium, magnesium, and copper during pregnancy resulted in a decreased risk of the offspring developing AD.	(42)
					(43)
	Breast milk	SCFA	Metabolomics (GC-MS)	Caprylate and acetate might negatively correlate with AD	(44)
AD & FA	Faeces	SCFA	Metabolomics (HPLC)	Decreased gut propionate, butyrate, and valerate in AD and FA	(45)
					(46)
	Serum	Lactate	Metabolomics (LC-MS)	Higher lactate levels have been suggested as a non-invasive marker for evaluating temporal alterations in cell stress and toxicity in AD and FA. Lactate levels can serve as a marker of the balance between oxidative and anaerobic metabolism.	(47)
	Faeces	Lactate	Metabolomics (Enzymatic analysis using a D-/l-lactic acid assay kit & HPLC)	Infants with eczema were found to have increased lactic acid levels at 26 weeks of age, suggesting a potential link between lactic acid levels and development of AD. In addition, there was a significant increase in Lactococcus, a lactic acid-producing bacterium, with the introduction of non-hydrolyzed cow's milk proteins in infants who successfully outgrew oral milk allergy without immunotherapy.	(48)
					(49)
FA	Faeces	Fecal microbiota	Bacterial 16S ribosomal RNA sequencing	Reduced *Ruminococcus, Bacteroides, Prevotella, and Coprococcus* in FA from infancy to school age. Reduced *Prevotellaceae* family in FA at 6 months.	(50)
					(51)
	Serum	Tryptophan metabolites, eicosanoids, plasmalogens, phospholipids	Metabolomics (UPLC-MS/MS & GC-MS)	Children with FA exhibited dysregulation of metabolites such as lysoplasmalogens, lysophospholipids, N2-acetyllysine, β/γ tocopherol, and sphingomyelins, which were uniquely altered compared to controls.	(52)
	Faeces	Acylglycerols	Metabolomics (LC-MS)	Significant enrichment of diacylglycerol was found in healthy twins compared to infants with FA.	(53)
	Cord plasma	Acylglycerols	Metabolomics (LC-MS)	Triacylglycerols of long carbon chains and multiple double bonds have been identified as potential novel predictive biomarkers for identifying high-risk children FA.	(54)
	Faeces	Bile acids	Metabolomics	Variations in primary bile acid biosynthesis and reduced levels of intestinal bile acid metabolites synthesized through the alternative pathway have been observed to differentiate subjects with persistent FA from healthy controls and those in remission from FA.	(55)
	Serum	PUFAs	Metabolomics (LC-MS)	A decline in omega-3 PUFAs levels was observed in resolving FA cases, while an increase occurred in persistent FA cases.	(56)

(GC-MS, gas chromatography-mass spectrometry; LC-MS, liquid chromatography-mass spectrometry; LC-ESI-MS/MS, liquid chromatography electrospray ionization tandem mass spectrometry; UPLC-MS/MS, ultra-performance liquid chromatography coupled with high resolution mass spectrometer; HPLC, high-performance liquid chromatography; PUFAs, polyunsaturated fatty acids; NMR, Nuclear magnetic resonance; MALDI-ToF MS, Matrix Assisted Laser Desorption Ionization-Time of Flight Mass Spectrometry).

## 2 Proteome

The study of the proteome, which is the set of proteins present in a cell at a certain time, is called proteomics, and remains challenging due to its dynamic nature (57). Proteomics usually encompasses multi-dimensional separation and protein identification by mass spectrometry (MS), followed by data analysis by bioinformatic tools. Regarding allergic diseases, proteomic approaches include the measurement of allergen-specific IgE and IgG antibodies, serum tryptase, “damage-associated molecular patterns (also called alarmins), as well as basophil and mast cell activation tests, among others. Some of these procedures are commonly used for diagnosis in clinical practice with more or less satisfactory outcomes depending on the type of allergy innovations in this field are needed to provide novel and robust protein biomarkers that contribute to a better understanding of the molecular mechanisms underlying allergic diseases. In this sense, recent technological advancements in next-generation proteomics, such as OLINK® PEA (Proximity Extension Assay) technology (OLINK Proteomics, Uppsala, Sweeden), enable multiplexing thousands of proteins using minimal sample amounts (58, 59). Therefore, it is a promising tool to improve stratification of patients and characterization of allergic diseases.

### 2.1 Proteome in AD

The AD proteome is still largely unexplored. Searching for “atopic dermatitis” in proteomic databases (8th November 2023), such as PRIDE [proteomics identifications database; (60)] or GPM [global proteome machine; (61)] retrieved 16 entries, and entries from 3 different studies, respectively (62–64). Twenty-seven AD-related proteins (retrieved on 1st September 2017) were previously reported in Uniprot (2, 65–67).

Regarding the IgE and IgG levels in AD, it is long known that children and adults with AD had high or very high serum IgE levels (68–74). More recently, Wollenberg et al. reviewed data on the efficacy of omalizumab, an anti-IgE biological, in AD and found them inconclusive, as many studies have reported varying degrees of efficacy of this biological drug. Five years earlier, Totté et al. described the association of AD severity with the IgG response against Staphylococcus aureus in young children.

A systematic review and meta-analysis about the correlation between AD severity and biomarkers previously identified serum thymus and activation-regulated chemokine (TARC/CCL17), a member of the T-helper 2 (Th2) chemokine family (TARC), attractant of Th2 effector cells, as the most reliable AD severity biomarker for both children and adults (75, 76). Further Th2-related chemokines have also emerged as potential biomarkers, including CCL18/PARC (pulmonary and activation-regulated chemokine), CCL22/MDC (macrophage-derived chemokine), CCL26/eotaxin-3, CCL27/CTACK (cutaneous T-cell-attracting chemokine), and lactate dehydrogenase (LHD), but additional research is needed (8, 75, 77–79).

Serum interleukin (IL)-13 and IL-22 levels are known to be higher in AD patients than in healthy controls (80). However, AD cytokine biomarkers are preferably assessed in the skin. Upregulation of thymic stromal lymphopoietin (TSLP), IL-4, IL-13, and IL-33, has been related to AD pathogenesis (80–83). Mouse models of AD have demonstrated the critical role of TSLP, which is released by keratinocytes upon epithelial damage, in inducing Th2 responses and AD pathogenesis (84–86). In addition, TSLP plays an important role in the differentiation of follicular T helper cells (T_FH_), which are critical players in humoral immunity, and AD severity in children (83, 87, 88). In addition, the proinflammatory cytokine IL-33, which belongs to the IL-1 inflammatory cytokine family, activates group 2 innate lymphoid cells (ILC2s) that induce the expression of IL-5 or IL-13. Moreover, IL-33 directly acts on keratinocytes by reducing the expression of junctional proteins, such as filaggrin and claudin-I, leading to skin barrier disruption (82). Similarly, IL-4 and IL-13 downregulate keratinocyte filaggrin expression (26, 89). *Pavel AB* et al. found considerably higher levels of inflammatory markers, such as proinflammatory interleukins (IL-1R1, IL-33), and metalloproteinases (MMP12) in lesional and non-lesional skin of moderate-to-severe AD patients (26).

Searching for biomarkers by non-invasive sampling approaches, *Holm* et al.*,* quantified 203 proteins in the vernix of 34 newborns, of which 18 children had developed AD at two years of age (24). The study showed that peroxiredoxin-2 and serpin A12 were present at higher levels in the AD group, and polyubiquitin-C and calmodulin-like protein 5 at lower levels when compared to healthy children. These four proteins had the highest impact on an orthogonal projection to latent structures-discriminant analysis (OPLS-DA) model. The authors concluded that the abundances of polyubiquitin-C and calmodulin-like protein 5, negatively correlated with AD development, are promising AD biomarker candidates.

When looking for reliable protein biomarkers, age at sampling is a key factor to take into account, since the proteome is dynamic over time. *Stockfelt* et al.*,* phenotyped 230 proteins in plasma prepared from umbilical cord blood, and blood collected at 1, 4, 18 months of age (and further) (25). They found that samples collected at 1 month of age were the most informative for the prediction of atopy later in childhood. Precisely, they found 27 proteins, including MHC class I molecules, interleukins and chemokines that were associated either with a lower risk, such as MHC class I proteins, IL-15 as well as TNFα-induced protein 3 (TNFAIP3) and Myeloid Derived Growth Factor (MYDGF), or a higher risk of developing atopy later in life, such as the interleukins IL-9 and IL-17, the chemokine receptor-10 (CCR10), the Growth Arrest and DNA Damage Inducible-α (GADD45A), as well as the proteases MPO and MMP9.

On the other hand, stratification of patients according to a cluster of several protein biomarkers, seems to be a promising approach in the elucidation of AD endotypes, as shown by studies from *Thijs JL et al*. and followed up by *Bakker DS et al*. (90, 91). In the first study, four serum biomarker-based clusters were identified and associated with different severity scores in a cohort of 193 patients. Three years later, with a different cohort of 143 AD patients, Bakker DS et al. identified four distinct patient clusters, three of them very similar to the previously identified. However, none of the studies could show a clear association between the clusters and atopic comorbidities or epidemiological variables, such as age. More studies are needed in this sense because the elucidation of AD endotypes might contribute to the development of personalized medicine and the better election of specific therapies in the management of AD.

### 2.2 Proteome in FA

Component resolved diagnosis or molecular diagnosis has led to a shift in the diagnosis of FA, improving accuracy, allowing the identification of complete amino acid sequences and IgE-binding epitopes of food allergens. These advances contribute to improved diagnosis and prognosis of FA, as well as safety assessment of foods and allergy testing (92, 93). Several immunological markers, such as specific IgE (sIgE), IgG4, sIgE/total IgE ratios or cytokines, have been monitored ever since in the course of FA. *Kukkonen* et al.*,* described that sIgE levels to Ara h 2 and Ara h 6 were associated with more severe reactions to peanut in a cohort of 102 patients (6- to 18-year-olds) with peanut sensitization and a high risk of suffering peanut allergy (94).

Compared to peanut and tree-nut allergies, milk and egg allergies have better outgrown outcomes in children (94). Cow's milk tolerance has been associated with elevated levels of cow's milk-specific IgG_4_ and an elevated IgG_4_/sIgE ratio in two independent studies (95, 96). However, in 2018, another study found no significant correlation between sIgE levels and milk tolerance in a cohort of 84 cow's milk allergic infants (6 months-3 years old) who tolerated baked milk in oral food challenge) (97). In the case of egg allergy, ingestion of baked egg together with decreased sIgE levels and increased egg-specific IgG4 were associated to the development of egg tolerance, while a Th1 cytokine profile and increased levels of IL-10 protein were associated to allergy resolution (98–101).

Biomarkers other than specific IgE and IgG antibodies are being studied in FA. Proteins or peptides released after immunological stimulation, degranulation, or cell damage or death, have been extensively described in the pathogenesis of asthma or AD (as discussed in the previous section). These molecules have been referred to as alarmins since 2006 and are increasingly considered in the study of the pathogenesis of FAs, as well as their potential role as biomarkers of the disease or possible therapeutic targets (102, 103). When damage occurs, due to inflammation or pathogen invasion, epithelial cells initiate a cascade of events encompassing the production of alarmins, such as TSLP, IL-33, and IL-25 (104). These signaling molecules activate innate lymphoid cells (ILC2) and Th2 cells, leading to the release of cytokines that promote a Th2-type immune response. For example, IL-5 and IL-13, produced by these alarmins, contribute to the recruitment and accumulation of eosinophils in the tissue and trigger the switch in IgE antibody production, respectively. IL-4, produced by Th2 cells, activates mast cells and basophils (105). Cutaneous infiltration of basophils may direct eosinophil recruitment and enhance food antigen sensitization. These observations emphasize the hypothesis that skin barrier disruption in children triggers transcutaneous sensitization and leads to the development of IgE-mediated FA (106).

Another type of alarmin is calprotectin, an immunomodulatory and antimicrobial protein found in mucosal epithelial cells, macrophages, and neutrophil cytoplasm (107). As its concentration in feces is six times higher than in plasma, it is considered a fecal biomarker and is well-established as a reliable biomarker of intestinal inflammation (108–110). Regarding FA, it has been proposed as a good diagnostic indicator of cow's milk protein allergy (CMPA) and shows promise for monitoring intestinal allergies due to its correlation with inflammation and immune responses to food antigens (111–114).

The heterogeneity of FA, with multiple triggers and confounding factors in the allergic history of patients, makes it necessary to continue the search for reliable and robust biomarkers and validate them for use in daily clinical practice.

## 3 Microbiome

The term microbiota refers to the living microorganisms inhabiting a defined environment or body site, such as the skin, the gut, the oral cavity, and the vagina. The term microbiome refers to “the collection of genomes from all the microorganisms” in a specific environment. This “also includes the microbial structural elements, metabolites, and environmental conditions” (22, 115, 116).

Initial colonization of the microbiota at the body's interface occurs at birth. During natural delivery, the mother provides the neonate with the founder commensals, derived from her vagina and faeces (117, 118). Breast milk feeding further helps shaping microbiota diversification by providing secretory IgA and prebiotic glycans that promote the expansion of specific species (119–121). In contrast, delivery by caesarean section provides the infants with the mother's skin-derived microbiota (122–124). A study found that caesarean section delivery predisposed to the development of FA but not AD in early childhood (125). In turn, epidemiological studies have linked perinatal (pre- and post-) antibiotic use to the appearance of AD and cow's milk allergy in infants (126–128). Whether early disruption of the microbiota directly affects future risk of allergic diseases remains elusive.

### 3.1 Microbiome in AD

Skin microbiota composition in infants shifts over time being distinct from that observed later in life. Therefore, age affects skin microbiome composition, together with the birth delivery mode and maternal commensals (27, 129). Longitudinal studies found alterations in skin microbiota that predate AD onset. Two-day-old infants have site-specific differences in their skin microbiomes that might influence the future development of AD. Precisely, the abundance of *Staphylococcus* species was higher on the extremities, whereas *Gemella* and *Propionibacterium* species were more abundant on facial sites (27). Further evidence showed that 12-month-old infants with affected skin had significantly less commensal *Staphylococci* at month 2 when compared with unaffected infants (27, 130). Thus, commensal *Staphylococci* might confer protection against AD development. In a prospective birth cohort study, *Staphylococcus aureus'* prevalence was higher on the skin of 3-month-old infants who developed AD later, as compared with age-matched, non-atopic infants. Moreover, at AD onset, infants with *S. aureus* on their skin were younger than those without it (30). Several genomic studies confirmed *S. aureus* abundance and that of other coagulase-negative *Staphylococci* (CoNS) to increase in AD, whereas *Streptococcus, Propionibacterium, Acinetobacter, Cutibacterium,* and *Corynebacterium* genera are typically present in the homeostatic skin (28, 29, 131–134). These data suggest that alterations in skin colonization may contribute to AD onset in early life. Currently ongoing, the “Munich Atopic Prediction Study” collects information on pregnancy, parental exposures to potential allergens, environmental factors, child development, and acute or chronic diseases of both children and parents together with microbiome analyses from stool and skin swabs, and clinical examination by trained dermatologists at 2 months after birth and every 6 months thereafter. Results from this study will certainly be useful for the identification of AD biomarkers (135).

Studies in mice showed that the establishment of a homeostatic skin microbiota are preferential in early life, and likely to be more significant in the skin than in other tissues (136). When neonatal mice are colonized by *S. epidermidis,* a CoNS commensal, a large proportion of Tregs specific for *S. epidermidis* develops, ensuring homeostasis to this microbe. In contrast, delaying exposure to *S. epidermidis* abrogates its protective effect and promotes skin inflammation (137).

Prominent research shows the impact of the gut microbiome on the skin microbiome and the development of skin diseases (138, 139). This is referred to as the gut-skin axis (140). *Wang* et al.*,* found a reduced diversity in the faecal microbiota of infants with AD during the first 18 months of life (31). Moreover, *Penders* et al.*,* observed an association between the colonization with *Clostridium* species at 5 and 13 weeks of age with an increased risk of AD in the subsequent 6 months of life (33). In turn, *Marrs* et al.*,* described that a greater abundance of *Clostridium sensu stricto* was associated with AD in 3-month-old breastfed infants (32). In contrast, recently, *Park* et al. found low levels of *Clostridium* and *Akkermansia* and high levels of *Streptococcus* in children with persistent AD. Moreover, the relative abundance of *Streptococcus* positively correlated with AD score (SCORAD), whereas that of *Clostridium* negatively correlated with SCORAD. (34). The findings of these two studies may seem contradictory. However, the different experimental approaches may account for their different findings. *Park* et al. did not define any correction for confounding factors such as introduction to solid foods or mode of delivery, and they collected faecal samples at 6 months of age, particularly when *Marrs* et al. found the larger differences in gut microbiota composition due to diet. Moreover, the definition of the *Clostridia* species also differed in these two studies.

Understanding the factors modulating skin and gut microbiomes is essential for identifying predictive AD biomarkers and maintaining a homeostatic skin.

### 3.2 Microbiome in FA

The gut microbiota is a major ecosystem, which changes in response to environmental factors such as diet, pathogens, and antibiotic treatment, significantly influencing immune system maturation through dendritic cell–mediated regulation (124, 141, 142). Alterations in the gut microbiota composition together with decreased levels of Tregs have been found in subjects with rhinitis, asthma, AD, and FA to peanuts, eggs, or cow's milk (22, 50, 124, 143–145). The amount of Tregs in the gut seems to associate to specific genera of bacteria; therefore, this could be a mechanism by which the gut microbiota modulates FA course. Protective effects against FA have been linked with genera such as *Lactobacillus, Bifidobacterium, Faecalibacterium, Akkermansia, Staphylococcus,* and *Clostridium* (51, 146–148)*.* SCFA-producing bacteria from phylum Firmicutes, including *Lactobacillaceae*, *Ruminococcaceae* and *Lachnospiracea* families, actively contribute to regulating intestinal immune responses, by enhancing mucin production, improving barrier integrity, and preventing the colonization of harmful strains (149–152). On the contrary, certain genera, such as *Clostridium, Enterococcus, Klebsiella*, and *Enterobacter*, have been related to a higher likelihood of developing FA (146, 147, 153).

*Mera-Berriatua* et al.*,* analysed the faecal microbiota of 34 cow's milk allergic infants vs. 16 non-allergic controls (51). They found an increased frequency of the *Prevotellaceae* family in control and formula-fed infants compared to allergic and hydrolysate-fed infants, respectively. However, since samples were collected at only one timepoint (6 months old) when cow's milk allergy was already established, they could not discern whether differences were due to the infants’ allergy or their diet. When analysing the gut microbiota of the mothers, they did not find anymicrobial signatures predisposing to FA and transmitted by the mothers. *Abdel-Gadir* et al.*,* analysed the faecal microbiota of 56 infants with FA and 98 controls at different times (154). They found that infants with FA presented a dysbiotic faecal microbiota composition that evolved over time. Therapy with *Clostridiales* or *Bacteroidales* suppressed FA in mice. Recently, early inoculation with certain *Clostridium* species was reported to decrease IgE levels in adulthood. Conversely, 3-week-old infants that presented higher proportions of *Clostridium difficile* than *Bifidobacterium* had higher chances of being allergic to food and aeroallergens (155).

These findings underscore the intricate relationship between gut microbiota, immune function, and FA course. Despite advances in this field, it is still unknown whether a dysbiosed gut microbiota triggers FA or is the disease itself the one altering microbiota composition and functionality.

## 4 Metabolome

Metabolites are the final products of cellular activity and significantly impact immune responses. Therefore, the identification and analysis of metabolic biomarkers is essential to improve our understanding of AD and FA. The Human Metabolome Database [HMDB; (156)] offers an efficient electronic resource readily accessible for comprehensive information on human metabolites. It includes links to various databases, along with numerous tools for structure and pathway visualization. Additionally, this database facilitates guidance on diseases wherein the target metabolite can act as a biomarker.

### 4.1 Metabolome in AD

The skin of mammals, especially the epidermis, contains extensive amounts of lipids. These lipids, including ceramides, cholesterol, and free fatty acids, are integral epidermal barrier components (140, 157). Lipid alterations in the outer skin layer led to barrier impairment and eventually to AD. The alteration of epidermal ceramide composition also promotes inflammatory and allergic events in infants with AD (36). Moreover, reduced levels of ceramides associate with *S. aureus* colonization in AD patients (35). A recent study on newborns showed that at the age of 2 months, children with future AD had reduced protein-bound ceramides and increased unsaturated sphingomyelin species in the skin. This study demonstrated a significant predictive power for AD in 2-month-old children. Precisely, TSLP, at the age of 2 months was predictive of the onset of atopic dermatitis by the age of 24 months with an odds ratio (OR) of 4.1 (95% CI, 1.7–10.1). When combined with family history of atopic diseases, the predictive power of TSLP level rose to the OR of 6.0 (with 95% CI of 2.3–15.8). Additionally, the combination of high TSLP, high 24:1-SM, and low O30:0(C20S)-CER had the highest OR of developing AD by the age of 24 months at 29.6 (95% CI, 4.9–179.7) (37).

Moreover, alterations in the fatty acid composition of plasma [decreased levels of omega-3 polyunsaturated fatty acids (PUFAs) and increased levels of omega-6 PUFAs] have been observed in infants who later develop AD. Furthermore, the findings suggest that adjusting the diet to include more omega-3 fatty acids, particularly through fish oil supplementation during pregnancy and lactation, could effectively restore this balance and potentially reduce the risk of AD in infants (40). However, information on other relevant metabolites in adult AD, such as sphingosine 1-phosphate and acylcarnitines, is lacking in children (158). These data suggest that an in-depth analysis of cutaneous lipid profile could aid in the early diagnosis and effective management of AD (159).

As previously said, the gut-skin axis is important in AD. Gut microbiota generates numerous metabolites, which can permeate the circulatory system and reach remote body sites (160). SCFA, including acetate, propionate, butyrate, and valerate, are byproducts of intestinal microbiota fermentation of fibre (44, 149, 161–163). A distinct SCFA profile has been identified in AD patients. Therefore, nutrition plays a key role. Atopy in children has been associated with deficiencies of micronutrients such as iron (164, 165). Iron deficiency leads to immune cell activation (166). A study reported that when mothers were supplemented for both iron and folic acid, their children had a 4-fold decreased risk for AD (42). In a systematic review, *Venter* et al.*,* found that maternal intake of beta-carotene, vitamin E, zinc, calcium, magnesium, and copper during pregnancy resulted in a decreased risk of AD for the offspring. Supplementation with omega-3 PUFAs in pregnant women has been shown to reduce infant sensitization to egg, and the risk and severity of atopic dermatitis in the first year of life, especially in children of mothers with a low habitual intake of omega-3 PUFAs (40, 43, 167–169). Several studies provided preliminary evidence of a protective effect of prenatal vitamin D supplementation on the likelihood of childhood AD. Notably, infants with AD had a significant vitamin D deficiency and insufficiency, which correlated with the severity of IL-17A-dependent AD (170). Additionally, prenatal maternal depression and anxiety were reported to increase the risk of AD in the offspring by decreasing the ratio of placental glutathione to glutathione disulfide (38). This implies that oxidative stress plays a part in the pathogenesis of AD. In fact, thiol-disulfide balance has been proposed as a biomarker of childhood AD (39). These findings highlight the importance of diet in the maternal period and early life, as alterations of the metabolome in AD infants have been in correlation to the gut microbiota.

### 4.2 Metabolome in FA

Various metabolites have been recognized as potential biomarkers for FA. *Crestani* et al.*,* described reduced sphingolipids and ceramides in children with FA, indicating a potential association between altered gut microbiota and immune function. Children with a history of anaphylaxis and multiple allergies display alterations in tryptophan metabolites, including decreased levels of metabolites in the kynurenine and serotonin pathways, and increased levels in the indole pathway. Additionally, changes in eicosanoids, plasmalogens, and fatty acids have been observed. Children with FA exhibited unique dysregulation of lysoplasmalogens, lysophospholipids, N2-acetyllysine, β/γ tocopherol, and sphingomyelins, compared to controls (52). A recent prospective study established the relationship between newborn lipid profiles and the development of FA. Cord triacylglycerols of long carbon chains and multiple double bonds were identified as potential early-life biomarkers for high-risk infant FA (54). Another study described a significant enrichment of diacylglycerol in healthy twins compared to those with FA (53). Additionally, children with FA exhibited a significant increase in sphingolipids and a decrease in acylcarnitines when compared to healthy controls. Interestingly, at the time of diagnosis, individuals with resolving FA displayed elevated omega-3 PUFAs levels and decreased platelet-activating factors in comparison to those with persistent FA. However, over time, a decline in omega-3 PUFAs levels was observed in resolving FA cases, while an increase occurred in persistent FA cases (56). Omega-3 metabolites, derived from eicosapentaenoic acid (EPA) and docosahexaenoic acid (DHA), play a crucial role in this context. In individuals with resolving FA, high levels of hydroxydocosahexaenoic acid (HDoHE) were observed, while levels of the pro-resolving mediators RvD1 and RvD2 were low. This may be attributed to the utilization of 17-HDoHE during the production of resolvins. Such a process could reduce IgE production by human B cells and suppress the differentiation of naive B cells into IgE-secreting cells, which are relevant in the context of allergies. Additionally, RvD1 has been reported to modulate allergic airway responses by reducing eosinophils and pro-inflammatory mediators. Furthermore, the association between HDoHE levels and the PAK5 gene, previously linked to atopy and psoriasis, supports their relevance to allergic diseases. The study suggests that HDoHEs and D-series resolvins may play a significant role in the pathophysiology of FA and FA resolution, potentially serving as markers for FA resolution. However, it is important to note that studies on omega-3 exposure in childhood allergic diseases have produced inconsistent results, highlighting the complexity of these processes.

Distinct patterns of bile acids, amino acids, steroid hormones, and SCFA were found in individuals with FA. At 3–6 months and/or 1 year of age, reductions were observed in bile acids, amino acids, and steroid hormones. Conversely, individuals with FA exhibited elevated levels of diacylglycerols at 3–6 months. Variations in primary bile acid biosynthesis and reduced levels of intestinal bile acid metabolites synthesized through the alternative pathway have been described to distinguish subjects with persistent FA from those in FA remission (55). Furthermore, the microbiome's production of SCFA is crucial for gut health and immune modulation. Changes in the microbiome that lead to alterations in bile acid metabolism and SCFA production may impact the persistence or remission of food allergies. Precisely, lower levels of propionate, butyrate, and valerate have been associated with FA in infants (45, 46).

Although there is limited information on immune metabolism in AD and FA, both conditions have been associated with impaired barrier function of epithelial cells, characterized by a predominantly glycolytic phenotype and mitochondrial dysfunction (171, 172). Increased lactate levels, resulting from increased lactic acid bacteria, have been proposed as a non-invasive marker for assessing temporary changes in cellular stress and toxicity in AD and FA (41, 47–49).

**Figure 1:**
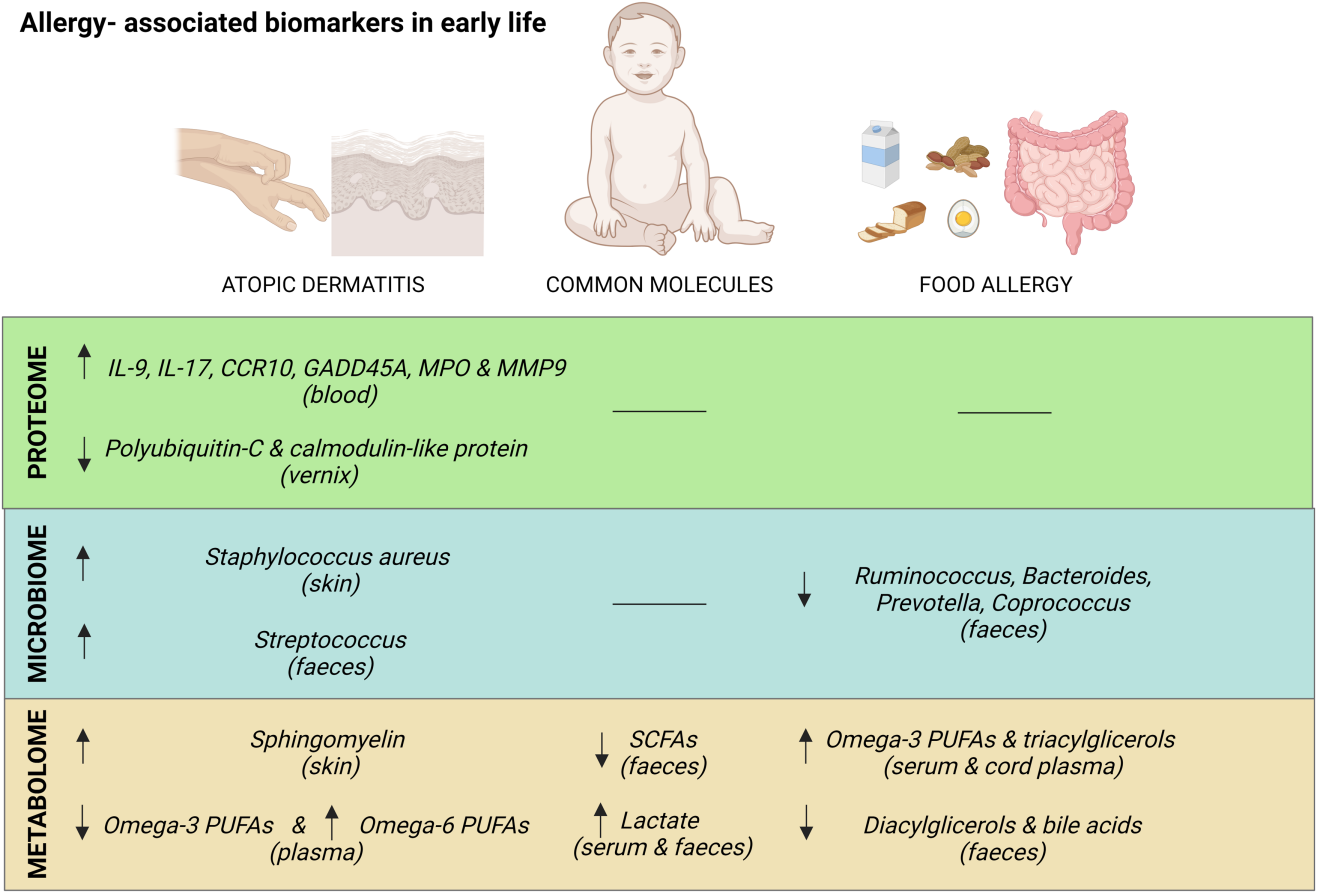
Summary of major common and exclusive biomarkers for atopic dermatitis and food allergy in early life. Created with Biorender.com.

Figure 1 summarizes the major biomarkers identified by omics techniques associated with AD and/or FA in early life.

## 5 Conclusions

The identification of potential biomarkers in early life could improve the diagnosis, monitoring, and management of AD and FA. Exploring the relationship between proteomics, microbiota and metabolomics is crucial, as some alterations in the microbiome can induce changes in the host. For example, common microbiota-derived metabolites such as SCFA and lactate, along with the presence of certain bacteria in faecal samples such as the genus *Streptococcus*, may play a role in shaping the host immune response. The integration of data from various omics technologies coupled with advances in statistical and computational techniques, is expected to enable the application of precision medicine and targeted therapy in allergic patients within the next decades, allowing the classification of heterogeneous diseases like FA and AD into endotypes and phenotypes. There is therefore an urgent need of extensive clinical and basic research to identify biomarkers in AD and FA specific for different allergic phenotypes.
